# Cost-Effectiveness Analysis of Type 2 Diabetes Mellitus (T2DM) Treatment in Patients with Complications of Kidney and Peripheral Vascular Diseases in Indonesia

**DOI:** 10.3390/healthcare9020211

**Published:** 2021-02-16

**Authors:** Akhmad Priyadi, Hikmat Permana, Ahmad Muhtadi, Sri A. Sumiwi, Rano K. Sinuraya, Auliya A. Suwantika

**Affiliations:** 1 Department of Pharmacology and Clinical Pharmacy, Faculty of Pharmacy, Universitas Padjadjaran, Bandung 40132, Indonesia; akhmadpriyadi@yahoo.com (A.P.); a.muhtadi@unpad.ac.id (A.M.); sri.adi@unpad.ac.id (S.A.S.); r.k.sinuraya@unpad.ac.id (R.K.S.); 2 Faculty of Pharmacy, Universitas Bhakti Kencana, Bandung 40164, Indonesia; 3 Department of Internal Medicine, Faculty of Medicine, Universitas Padjadjaran, Bandung 40132, Indonesia; hikmat.permana@unpad.ac.id; 4 Center of Excellence in Higher Education for Pharmaceutical Care Innovation, Universitas Padjadjaran, Bandung 40132, Indonesia; 5 Center for Health Technology Assessment, Universitas Padjadjaran, Bandung 40132, Indonesia

**Keywords:** blood glucose reduction, *BPJS Kesehatan*, ICER, perspective, healthcare provider, payer

## Abstract

Type 2 diabetes mellitus (T2DM) is a chronic disease with high-cost treatment. This study aimed to analyze the cost-effectiveness of T2DM treatment in hospitalized patients with complications of kidney and peripheral vascular disease (PVD) in Indonesia by focusing on patients of Health Social Security Agency (*BPJS Kesehatan*). An observational study was applied by collecting data retrospectively from patients’ medical record at the biggest public hospital in West Java Province, Indonesia. Two perspectives of payer and healthcare provider were applied to estimate the treatment cost. We considered following inclusion criteria: (i) Hospitalized T2DM patients without complication, with complications of kidney and PVD during 2014–2017; (ii) member of *BPJS Kesehatan*; (iii) >18 years old patients; and (iv) patients with complete medical record data. The results showed that the majority patients were female (56.72%), 45–64 years old (69.40%), and had a length of stay at 4–10 days (54.48%). The greatest contributions in the total treatment cost were found to be hospital room, medical services and medicines for the treatment of T2DM without complications, with complications of kidney and PVD, respectively. From the perspective of payer, the incremental cost-effectiveness ratios (ICERs) of T2DM treatment with complications of kidney and PVD would be IDR 215,723 and IDR 234,591 per 1 mg/dL blood glucose reduction, respectively. From the perspective of healthcare provider, the ICERs of T2DM treatment with complications of kidney and PVD would be IDR 166,289 and IDR 681,853 per 1 mg/dL blood glucose reduction in both perspectives (1 US$ = IDR 13,451). In a comparison with T2DM without complication, reducing 1 mg/dL blood glucose in T2DM treatment with complication of PVD would require higher cost than in T2DM treatment with complication of kidney from both perspectives.

## 1. Introduction

The prevalence of diabetes mellitus (DM) in Indonesia was estimated to be 8.6% of the total population, which placed this country in the fourth rank after India, China and America [1,2,3]. The International Diabetes Federation (IDF) has predicted the number of DM patients in Indonesia would increase from 9.1 million in 2014 to 14.1 million in 2030, which is linear with the result of Indonesia Basic Health Research in 2013 that confirmed the number of DM patients (>15 years old) in Indonesia at 12.2 million [4,5]. In general, there are two types of DM: Type 1 (T1DM) and type 2 (T2DM). In T1DM, β-pancreatic cells fail to secrete insulin that may lead the production of insulin to be very low [6,7,8,9,10]. While, T2DM is characterized by several disorders, such as impaired insulin secretion, insulin resistance (e.g., in muscles, liver, and adipose), and excessive secretion of glucagon-like-peptide-1 [11,12]. In a comparison with healthy people, T2DM patients are risky to have kidney failure (17 times higher) and diabetic ulcers (50 times higher) [13,14]. In Indonesia, the annual incidence of kidney failure was reported to be 200–250 cases in 1 million population [15,16,17]. Another most frequent case of T2DM complication is diabetic ulcer [18], which can cause up to 50% of non-pneumatic lower limb amputations [18,19]. The mortality and amputation rates in Indonesia were estimated to be 16% and 25%, respectively. In addition, approximately 14.3% and 37% of patients would die after one-year and three-year amputation, respectively [20].

Since T2DM is a lifetime and high-cost disease, its economic burden is evident. The economic burden of DM in Indonesia was estimated to be $1.27 billion in 2020 [21]. This burden was predicted to be much higher in the next decade since the number of DM patients in the world would increase from 171 million in 2000 to 366 million in 2030 [22]. In developing countries, the number of DM patients was estimated to increase 246% from 115 million to 284 million patients [23]. In Indonesia, this economic burden would be worsened by the limited access to healthcare centers [24]. A previous study also highlighted that complications have a significant impact on the costs of managing T2DM in Indonesia and confirmed that a comprehensive strategy could potentially reduce national healthcare expenditure [25].

Since the implementation of national health insurance system in 2014, the Indonesian National Healthcare Insurance (*BPJS Kesehatan*) has launched a chronic disease management program (*Prolanis*), which provides a comprehensive treatment for patients, including T2DM patients, to improve their quality of life [26]. To evaluate the effectiveness of T2DM treatment in Indonesia, it is necessary to conduct an economic evaluation by considering clinical and cost parameters. This study aimed to analyze the cost-effectiveness of T2DM treatment in patients with complications of kidney and peripheral vascular disease (PVD) in Indonesia by focusing on Health Social Security Agency (*BPJS Kesehatan*) patients and taking two different cost perspectives into account. This type of analysis can be used to assess whether the additional benefits of the treatment are commensurate with its additional costs [27,28,29].

## 2. Methods

An observational study was applied by collecting data retrospectively from patients’ medical record at the biggest public hospital in West Java Province, as the most populous province in Indonesia (total population: 49,316,712; working age group: 45%; sex: 51% of male and 49% of female) with the highest prevalence of T2DM (8%) [30,31]. As the implementation of Indonesian constitutional duty to provide social security for all citizens, the government of Indonesia initiated the national health insurance program in 2014, which was operated by BPJS Kesehatan. In this study, we considered following inclusion criteria: (i) Hospitalized T2DM patients with no complications, complications of kidney and PVD in a period of 2014–2017; (ii) member of *BPJS Kesehatan*; (iii) patients in the age group of >18 years old; and (iv) patients with complete medical record data. We excluded patients who died and had no clinical improvement.

**Figure 1:**
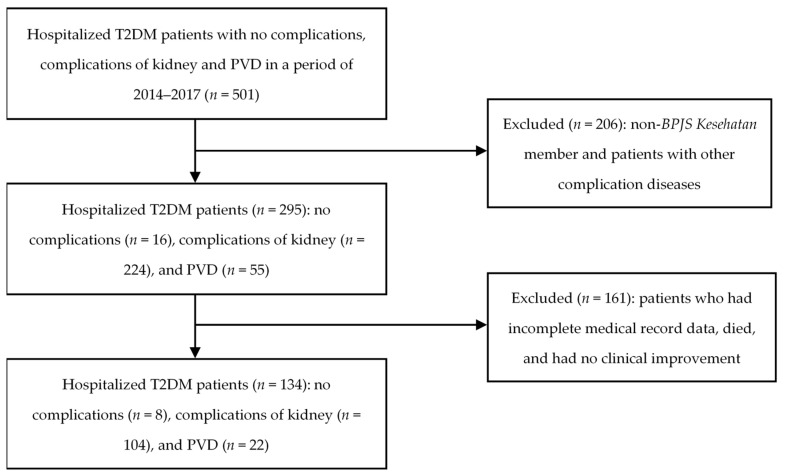
Flowchart of patient selection.

In a period of 2014–2017, we found 501 hospitalized T2DM patients with no complications, complications of kidney, and PVD. In this stage, we excluded 206 patients since they were not *BPJS Kesehatan* members and had other complication diseases. From 295 patients, we further excluded 161 patients who had incomplete medical record, died, and had no clinical improvement. We considered only 134 patients who met inclusion criteria, which consisted of 8, 104, and 22 patients without complications, with complications of kidney and PVD, respectively (see Figure 1).

In a comparison with T2DM treatment without complications, we estimated the incremental cost-effectiveness ratios (ICERs) of T2DM treatment with complications of kidney and PVD by considering parameters of cost and clinical effectiveness. We calculated ICER by dividing the difference in total cost by the difference in total effectiveness.

(1) $$\mathrm{ICER}\;=\;\frac{{\mathrm{Cos}t}_{\mathrm{with}\;\mathrm{complication}}{\;-\;\mathrm{Cos}t}_{\mathrm{without}\;\mathrm{complication}}}{{\mathrm{Effectiveness}}_{\mathrm{with}\;\mathrm{complication}}{\;-\;\mathrm{Effectiveness}}_{\mathrm{without}\;\mathrm{complication}}}$$

For hospitalizations due to T2DM complications, the following outcome measures were considered: Blood glucose reduction that was measured during hospitalization, length of stay, and total hospitalization costs. In particular, total hospitalization costs in this study were considered in two perspectives: Healthcare provider and payer. In the perspective of healthcare provider, we only considered direct medical costs, such as hospital room, doctors’ fee, medicines, laboratory tests, blood transfusion, and other medical services. All costs of hospitalized T2DM patients in this perspective were derived from medical record data. In the perspective of payer, we considered all costs covered by *BPJS Kesehatan*, as the third-party payer. All costs of hospitalized T2DM patients in this perspective were derived from the tariffs of Indonesia case-based groups (INA-CBGs). Descriptive statistics were applied to describe baseline characteristics of selected patients and all monetary values were reported in Indonesian Rupiah (IDR). To calculate the total cost, a discount rate of 3% was applied.

## 3. Results

From 134 patients who met inclusion criteria, we found that the majority gender and age group of patients are female (*n* = 76 and 45–64 years old (*n* = 93), respectively. In particular, the majority of patients had a length of stay at 4–10 days (*n* = 73) and kidney disease complications (*n* = 104). Information about patients’ characteristics can be seen in Table 1. The average total costs per patient in a period of 2014–2017 were estimated to be IDR 8,629,930; IDR 10,874,833; and 31,472,019 for the treatment of T2DM without complication, T2DM with complications of kidney, and PVD, respectively (see Table 2). In a comparison with other components in direct medical costs, the results showed that the greatest contributions in the total treatment cost were found to be hospital room (50.11%), medical services (28.18%), and medicines (29.07%) for the treatment of T2DM without complications, T2DM with complications of kidney and PVD, respectively (see Table 3).

In a comparison with T2DM treatment without complication, we estimated the incremental cost-effectiveness ratios (ICERs) from the perspective of payer would be IDR 215,723 and IDR 234,591 per 1 mg/dL blood glucose reduction for the treatment of T2DM with complications of kidney and PVD, respectively. In general, there were significant differences (*p*-value < 0.05) in total treatment cost between T2DM without complication, with complications of kidney and PVD during 2014–2017 (see Table 4).

From the perspective of healthcare provider, we estimated the ICERs would be IDR 166,289 and IDR 681,853 per 1 mg/dL blood glucose reduction for the treatment of T2DM with complications of kidney and PVD, respectively. In particular, there were significant differences (*p*-value < 0.05) in total treatment cost between T2DM without complication, with complications of kidney and PVD during 2016–2017 (see Table 5).

## 4. Discussion

The results showed that the majority patients were female (56.72%) and 45–64 years old. These results of this study are similar with the results of previous studies, which confirmed that population over 45 years old have a higher risk of T2DM since increasing of age will decrease physical activity, cause the glucose metabolism abnormal, increase glucose induction in insulin secretion, and insulin resistance [32,33]. A study by Chia et al. also mentioned that the presence of DM doubled the risk for CVD in men and tripled it in women, according to the Framingham and the Multiple Risk Factor Intervention studies [33]. In particular, the majority of patients had a length of stay (LoS) at 4–10 days (54.48%). It has been known that LoS is an important indicator to determine the effectiveness of T2DM treatment and it is related to the cost of care. The less LoS is associated with the more effective and efficient hospital services. In T2DM treatment, it has correlations with the knowledge of patients to deal with their lifetime disease [34,35]. The results of this study also showed that the majority of T2DM patients had kidney disease complications (77.61%), which is in line with a previous study in 2007 that showed the high prevalence (32%) of T2DM patients in Japan with microalbuminuria [36]. Another previous study in 2006 also confirmed that about 36% and 28% of T2DM patients in the US suffered from albuminuria and had kidney problems, respectively [37].

Our study gives some useful insight into the cost-effectiveness analysis of T2DM treatment in Indonesia by taking two different perspectives into account, but we should highlight several limitations of this study. Firstly, we obtained data from administrative database in the healthcare provider, which relied on its accuracy and its completeness of the patients’ records. Secondly, we did not consider the cost of side-effects in all treatments. In a lot of cases, side-effects due to treatment of T2DM-related complications are evident. Thirdly, unlike cost, a discount rate of health outcome was not applied in this study since we assumed that health outcome in our study would be less desirable with time.

To our knowledge, this is the first study to analyze the cost-effectiveness of T2DM treatment in patients with complications of kidney and PVD in Indonesia. Hence, it has several novel findings. The average total costs per patient for the treatment of T2DM with complications of kidney and PVD were reported to be 126.01% and 364.68% higher than T2DM without complications, respectively. The greatest contributions in the total treatment cost were found to be hospital room (50.11%), medical services (28.18%), and medicines (29.07%) for the treatment of T2DM without complications, with complications of kidney and PVD, respectively. These results strengthen the result of a study on assessing the impact of complications on the direct medical costs of T2DM in Indonesia, which confirmed that 84.35% T2DM outpatient patients had at least one complication of disease with an average treatment cost of $774.37 per patient for a six-month treatment [25]. The more complications will be linear with the higher treatment cost [38]. In addition, these particular costs of T2DM treatment with complications were associated with pharmacological treatment, diagnostic, and therapeutic procedures [25,38].

In a comparison with T2DM treatment without complications, we estimated the ICERs of T2DM treatment from the perspective of payer would be IDR 215,723 and IDR 234,591 per 1 mg/dL blood glucose reduction for the treatment of T2DM with complications of kidney and PVD, respectively. From the perspective of healthcare provider, the ICERs of T2DM treatment would be IDR 166,289 and IDR 681,853 per 1 mg/dL blood glucose reduction for the treatment of T2DM with complications of kidney and PVD, respectively. The results showed that reducing 1 mg/dL blood glucose in T2DM treatment with complication of PVD required higher cost than in T2DM treatment with complication of kidney from both perspectives. The ICERs in this cost-effectiveness analysis can be interpreted to find out the additional costs in one unit of effectiveness and to provide several alternative interventions by considering funding availability, specifically from the perspective of payer [30,39,40]. To gain more cost-effective T2DM treatment with complications, it is necessary for the government of Indonesia to intensify prevention strategies. A systematic review study by Siegel et al. on the cost-effectiveness of interventions to manage DM highlighted that there were several interventions with strong evidence to prevent T2DM complications, such as through screening for undiagnosed T2DM, managing risk factors, and early treatment interventions [41]. Screening for T2DM has been proven to be a cost-effective intervention. A systematic review study by Najafi et al. on the cost-effectiveness of T2DM screening concluded that screening is cost-effective to prevent T2DM with the ICER in a range from $516 to $126,238 per quality-adjusted life year (QALY). Siegel et al. also mentioned that screening every three years for the US population without DM had strong evidence of being very cost-effective at $7898/QALY, compared with no screening [42]. We also found strong evidence that managing DM risk factors through diabetes self-management education (DSME) for individuals with diabetes, compared with usual care, was very cost-effective. A randomized-controlled trial study on the cost-effectiveness of DSME highlighted that DSME was a promising investment and had a potential to be a cost-saving intervention [43]. Regarding early treatment interventions, we found strong evidence that early T2DM treatment with the use of telemedicine could be cost-effective [41,44]. Learning from the experience of other countries on managing risk factors to prevent T2DM complications, these interventions are promising to be included in the benefit package of social insurance in Indonesia.

## 5. Conclusions

The majority of patients in this study had a LoS at 4–10 days. Since LoS is closely related to the total treatment cost of T2DM, we investigated the greatest contributions in the total cost, which were found to be hospital room, medical services and medicines for the treatment of T2DM without complications, with complications of kidney and PVD, respectively. Despite the fact that the cost-effectiveness values of T2DM treatment with complications of kidney and PVD varied in different perspectives, reducing 1 mg/dL blood glucose in T2DM treatment with complication of PVD would require higher cost than in T2DM treatment with complication of kidney from the perspective of healthcare provider and payer.

## Figures and Tables

**Table 1:** Patients’ characteristics.

Category	Sub-Category	Total	%
Gender	Male	58	43.28%
	Female	76	56.72%
Ages (years)	18–44	10	7.46%
	45–64	93	69.40%
	≥65	31	23.14%
Length of stay (days)	1–3	14	10.45%
	4–10	73	54.48%
	>10	47	35.07%
Types of T2DM complications	Without complication	8	5.97%
	Kidney disease	104	77.61%
	PVD	22	16.42%

**Table 2:** Direct medical costs of T2DM treatment in Indonesian Rupiah (IDR) (1 US$ = IDR 13,451).

Types of T2DM Complication	Year	Cost *						
		Hospital Room	Doctors’ Fee	Medicines	Laboratory Tests	Blood Transfusion	Medical Services	Total Cost
Without complication	2014	2,647,000	500,000	2,456,500	1,439,500	-	345,940	7,388,940
	2015	2,864,887	486,812	547,225	880,825	-	1,315,689	6,095,439
	2016	4,825,000	800,000	843,000	680,376	-	1,983,500	9,131,876
	2017	7,711,548	1,076,358	1,192,141	754,391	297,056	871,972	11,903,465
	Average	4,512,109	715,792	1,258,716	938,773	74,264	1,129,275	8,629,930
Complication of kidney disease	2014	1,606,250	237,813	1,657,331	1,634,793	223,750	2,577,721	7,937,657
	2015	1,948,082	288,436	2,047,165	1,834,698	151,364	2,277,201	8,546,946
	2016	2,424,766	509,571	2,716,532	2,651,687	856,325	3,364,893	12,523,774
	2017	4,048,111	451,043	3,106,726	2,556,657	455,057	3,873,361	14,490,955
	Average	2,506,802	371,716	2,381,939	2,169,459	421,624	3,023,294	10,874,833
Complication of PVD	2014	7,055,200	1,427,500	10,563,515	3,629,325	618,300	9,194,600	32,488,440
	2015	9,746,297	1,888,830	11,592,904	3,214,702	1,301,086	10,942,231	38,686,049
	2016	4,576,995	792,428	5,751,749	3,781,058	407,443	7,439,602	22,749,276
	2017	5,180,618	878,947	13,208,775	3,846,804	1,148,742	7,700,424	31,964,311
	Average	6,639,778	1,246,926	10,279,236	3,617,972	868,893	8,819,214	31,472,019

* Discounted.

**Table 3:** Direct medical costs of T2DM treatment in percentage.

Types of T2DM Complication	Year	Cost *						
		Hospital Room	Doctors’ Fee	Medicines	Laboratory Tests	Blood Transfusion	Medical Services	Total Cost
Without complication	2014	35.82%	6.77%	33.25%	19.48%	0.00%	4.68%	100.00%
	2015	47.00%	7.99%	8.98%	14.45%	0.00%	21.58%	100.00%
	2016	52.84%	8.76%	9.23%	7.45%	0.00%	21.72%	100.00%
	2017	64.78%	9.04%	10.02%	6.34%	2.50%	7.33%	100.00%
	Average	50.11%	8.14%	15.37%	11.93%	0.62%	13.83%	100.00%
Complication of kidney disease	2014	20.24%	3.00%	20.88%	20.60%	2.82%	32.47%	100.00%
	2015	22.79%	3.37%	23.95%	21.47%	1.77%	26.64%	100.00%
	2016	19.36%	4.07%	21.69%	21.17%	6.84%	26.87%	100.00%
	2017	27.94%	3.11%	21.44%	17.64%	3.14%	26.73%	100.00%
	Average	22.58%	3.39%	21.99%	20.22%	3.64%	28.18%	100.00%
Complication of PVD	2014	21.72%	4.39%	32.51%	11.17%	1.90%	28.30%	100.00%
	2015	25.19%	4.88%	29.97%	8.31%	3.36%	28.28%	100.00%
	2016	20.12%	3.48%	25.28%	16.62%	1.79%	32.70%	100.00%
	2017	16.21%	2.75%	41.32%	12.03%	3.59%	24.09%	100.00%
	Average	20.81%	3.88%	32.27%	12.03%	2.66%	28.34%	100.00%

* Discounted.

**Table 4:** Cost-effectiveness value from the perspective of payer.

Year	Types of T2DM Complication	Total Cost (IDR)	Blood Glucose Reduction (mg/dL)	*p*-Value
2014	Without complication	11,330,400	95	0.01639 *
	Complication of kidney disease	15,064,238	98	
	Complication of PVD	21,475,940	65	
2015	Without complication	11,031,545	121	0.01747 *
	Complication of kidney disease	14,813,909	84	
	Complication of PVD	21,377,659	169	
2016	Without complication	6,094,319	54	0.02735 *
	Complication of kidney disease	9,663,898	93	
	Complication of PVD	14,614,062	87	
2017	Without complication	9,832,741	15	0.00227 *
	Complication of kidney disease	10,396,008	64	
	Complication of PVD	12,256,473	98	
Average	Without complication	9,572,251	71	0.01453 *
	Complication of kidney disease	12,484,513	85	
	Complication of PVD	17,431,034	105	
ICER **	Complication of kidney disease	IDR 215,723		
		per 1 mg/dL blood glucose reduction		
	Complication of PVD	IDR 234,591		
		per 1 mg/dL blood glucose reduction		

* Significant difference (*p*-value < 0.05); ** In a comparison with T2DM treatment without complication.

**Table 5:** Cost-effectiveness value from the perspective of healthcare provider.

Year	Types of T2DM Complication	Total Cost (IDR)	Blood Glucose Reduction (mg/dL)	*p*-Value
2014	Without complication	7,388,940	95	0.097
	Complication of kidney disease	7,937,657	98	
	Complication of PVD	32,488,440	65	
2015	Without complication	6,095,439	121	0.11595
	Complication of kidney disease	8,546,946	84	
	Complication of PVD	38,686,049	169	
2016	Without complication	9,131,875	54	0.03434 *
	Complication of kidney disease	12,523,774	93	
	Complication of PVD	22,749,276	87	
2017	Without complication	11,903,465	15	0.04541 *
	Complication of kidney disease	14,490,955	64	
	Complication of PVD	31,964,311	98	
Average	Without complication	8,629,930	71	0.07219
	Complication of kidney disease	10,874,833	85	
	Complication of PVD	31,472,019	105	
ICER **	Complication of kidney disease	IDR 166,289		
		per 1 mg/dL blood glucose reduction		
	Complication of PVD	IDR 681,853		
		per 1 mg/dL blood glucose reduction		

* Significant difference (*p*-value < 0.05); ** In a comparison with T2DM treatment without complication.

## Data Availability

Data available on request due to restrictions.
